# Recurrence rates and risk factors in obscure gastrointestinal bleeding

**DOI:** 10.1002/deo2.70011

**Published:** 2024-09-09

**Authors:** Sachiyo Onishi, Takuji Iwashita, Yukari Tezuka, Kentaro Kojima, Jun Takada, Masaya Kubota, Takashi Ibuka, Masahito Shimizu

**Affiliations:** ^1^ First Department of Internal Medicine Gifu University Hospital Gifu Japan

**Keywords:** balloon endoscopy, capsule endoscopy, obscure gastrointestinal bleeding, recurrence, small intestine

## Abstract

Obscure gastrointestinal bleeding (OGIB), defined in 2010, involves bleeding from the GI tract that remains unexplained after standard diagnostic procedures. OGIB, which accounts for about 5% of all GI bleeds, poses diagnostic and management challenges, particularly due to the anatomical features of the small intestine. Advances in capsule endoscopy (CE) and balloon‐assisted endoscopy have improved the diagnostic and therapeutic landscape for small intestinal lesions.

**Objective:**

To determine the recurrence rate and identify risk factors for recurrence following diagnostic and therapeutic interventions using CE and balloon‐assisted endoscopy in patients with OGIB.

**Methods:**

A retrospective cohort study at Gifu University Hospital analyzed CE procedures for patients with OGIB from 2008 to 2022. Patients underwent CE with subsequent treatments based on the findings. Statistical analyses, including Kaplan‐Meier and Cox proportional hazards models, were used to estimate cumulative recurrence rates and identify recurrence risk factors.

**Results:**

Out of 417 patients, 65.2% had positive CE findings, leading to therapeutic interventions in 16.3% of cases. The cumulative recurrence rates at 12, 24, and 36 months were 4.3%, 9.0%, and 13.9%, respectively. Liver cirrhosis (hazard rate: 4.15, 95% confidence interval 1.88–9.18, *p* < 0.01) was identified as a significant risk factor for recurrence.

**Conclusions:**

A significant recurrence rate in OGIB patients, with liver cirrhosis being a major risk factor. Despite diagnostic and therapeutic advances, a comprehensive approach including careful follow‐up and consideration of risk factors is essential for management.

## INTRODUCTION

Obscure gastrointestinal bleeding (OGIB) was delineated in 2010 as a condition involving bleeding from the GI tract that persists or recurs, with no identifiable cause upon esophagogastroduodenoscopy (EGD) and colonoscopy (CS). OGIB is classified into overt and occult categories. The overt OGIB is marked by continuous or prior instances of melena or hematochezia, while occult OGIB is characterized by repeated or enduring anemia due to iron deficiency, and a positive fecal occult blood test result. OGIB accounts for approximately 5% of all GI bleeding incidents^1^,^2^ with an estimated 75% of these cases stemming from the small intestine^3^. Given its significant prevalence, the examination of the small intestine is important in the diagnosis and management of OGIB. Nonetheless, the extensive length of the small intestine and its minimal attachment to adjacent structures pose substantial challenges for endoscopic examination. Recent advancements in capsule endoscopy (CE) and balloon‐assisted endoscopy (BAE) have significantly enhanced the diagnostic and therapeutic capabilities of small intestinal lesions.

The diagnostic efficacy of CE and BAE in identifying the source of bleeding in cases of OGIB has been reported to be 62% for CE and 56% for BAE.^4^ The diagnosis of small intestinal lesions and the implementation of appropriate treatment have been associated with favorable clinical outcomes.^5^ Treatment modalities include endoscopic interventions using BAE, intravascular radiology (IVR), surgical procedures, and conservative management. The success rate of clinical treatments varies from 40% to 73% with endoscopic interventions^6^, ^7^, ^8^, ^9^ and reaches 71% for IVR,^10^ indicating significant effectiveness. However, approximately 10% of OGIB cases experience rebleeding or recurrence of symptoms, necessitating rehospitalization, blood transfusions, and further diagnostic and therapeutic interventions.^11^ Identifying risk factors for rebleeding and recurrence of symptoms could facilitate strategies such as meticulous follow‐up and the initiation of early or intensified treatment. Previous studies typically have limited sample sizes. In this study, we analyzed a relatively larger cohort size. Therefore, this study aims to determine the recurrence rate and identify risk factors for recurrence following diagnostic and therapeutic interventions using CE and BAE in OGIB cases.

## PATIENTS AND METHODS

### Study design and participants

This retrospective cohort study was conducted at Gifu University Hospital, analyzing a database including all CE procedures from November 2008 to December 2022. Inclusion criteria were patients who underwent an initial CE for OGIB. Exclusion criteria were as follows: 1) patients in whom the capsule did not progress beyond the upper intestine, preventing small intestine observation, and 2) patients in whom CE procedure was hindered by factors such as food residue, rendering CE visualization difficult.

### CE procedure and treatment strategy

Prior to CE, all patients were required to fast for 8 h. On the test day, patients were equipped with the necessary devices at the hospital, consumed an adequate volume of water, and swallowed a capsule endoscope (PillCam SB or SB2 Plus; Medtronic). Water intake resumed 2 h post‐ingestion, followed by light meals after 4 h, concluding the test approximately 8 h later. Patients were then questioned to check for capsule excretion; in cases of uncertainty, a plain abdominal X‐ray was conducted to confirm excretion. CE image interpretation was performed independently by two board‐certified gastroenterologists, both experts in analyzing CE findings, to ensure accuracy and determine the necessity for further examinations or treatments. These considerations were discussed during weekly board meetings attended by the CE instructor. Subsequent to CE, the indications for additional diagnostics or treatment were essentially determined based on guidelines,^12^ but in cases where treatment strategies are unclear, decisions are made at conferences based on the advice of a CE Instructor Certified by the Japanese Association for Capsule Endoscopy. Treatment decisions were made case basis by the attending physicians. BAE was typically offered for bleeding from mucosal defects, such as erosions, or polyps within the small intestine. Lesions deemed unsuitable for endoscopic intervention were surgically treated at a later date. Patients who underwent hemostasis procedures were typically followed up with CE within 3 to 6 months.

### Study definitions and evaluation criteria

Recurrence was defined as patients who, following the initial CE and clinical successful management, experienced overt bleeding or in cases where there is a progression of severe anemia necessitating further small bowel examination. Overt bleeding encompassed bloody stools, melena, or hematemesis, while significant anemia was quantified by a reduction of at least 2 g/dL from baseline hemoglobin (Hb) levels. Positive CE findings were defined as follows: 1) tumors potentially causing bleeding, 2) in the absence of tumors, mucosal defects (such as erosions or ulcers) or vascular lesions implicated in anemia, and 3) visible blood without identifiable sources.

Bleeding from areas other than the small intestine^1^: CE showed bleeding from the esophagus, stomach, and duodenum^2^; CE showed no abnormal findings, and colonoscopy revealed the source of the bleeding.

Assessments covered patient demographics, including age, gender, comorbidities, medication use, laboratory data, CE timing, findings, and hemostatic interventions. Noted comorbidities included hypertension, dyslipidemia, diabetes under oral management, ischemic heart disease (with a history of myocardial infarction or angina), cerebrovascular disease (including stroke or cerebral hemorrhage history), chronic heart failure, chronic kidney disease, chronic obstructive pulmonary disease, and liver cirrhosis. Medication evaluations included the use of prednisolone, nonsteroidal anti‐inflammatory drugs, low‐dose aspirin, antiplatelet drugs, and anticoagulants, defined as oral administration initiated at least one month before OGIB symptom onset.

### Study outcomes and statistical evaluation

The primary outcome was the cumulative recurrence rate of bleeding, with secondary outcomes including the success rate of hemostasis in treating OGIB and identifying recurrence risk factors. We have defined success as the absence of recurrence, which includes no new episodes of bleeding or progression of severe anemia.

Data were presented as mean ± standard deviation (SD) for continuous variables and as numbers and percentages for categorical variables. The Mann‐Whitney U test compared continuous variables, while Fisher's exact test compared categorical variables. Cumulative rebleeding rates were estimated using the Kaplan‐Meier method. Risk factors for rebleeding were evaluated through univariate and multivariate analyses using Cox proportional hazards models, with adjusted hazard ratios (HRs) and 95% confidence intervals. *p*‐Values of <0.05 were considered statistically significant. Statistical analyses were conducted using JMP Pro for Windows software (version 13).

## RESULT

### Patient background

A total of 417 patients with an average age of 65.2 years including 238 males (57.1%) were included in this analysis. The mean minimum Hb value was 8.9 g/dL. The type of OGIB was overt in 155 cases (37.2%) and occult in 262 cases (62.8%; Table 1). Table 2 lists the underlying diseases. Regarding oral medication use, 73 patients (17.5%) were on antiplatelet drugs, 63 (15.1%) on anticoagulants, 109 (26.1%) on NSAIDs, and 53 (12.7%) on prednisolone.

**TABLE 1:** Patient baseline clinical characteristics.

Variable	
Age, years, mean (SD)	65.2 (15.5)
Gender, *n* (%)	
Male	238 (57.1)
Female	179 (42.9)
BMI, kg/m^2^, mean (SD)	22.6 (9.3)
Minimum hemoglobin level, g/dL, mean (SD)	8.9 (2.5)
Albumin, g/dL, mean (SD)	3.2 (0.68)
Obscure GI bleeding type, *n* (%)	
Overt	155 (37.2)
Occult	262 (62.8)

Abbreviations: BMI, body mass index; GI, gastrointestinal; SD, standard deviation.

**TABLE 2:** Patients' underlying diseases and oral medications at baseline.

**Underlying disease, *n* (%)**	**History of myocardial infarction**	**51 (12.2)**
	Congestive heart failure	52 (12.5)
	Cerebrovascular accident	36 (8.6)
	Chronic obstructive pulmonary disease	19 (4.6)
	Chronic hepatitis	17 (4.1)
	Liver cirrhosis	40 (9.6)
	Diabetes mellitus	34 (8.2)
	Chronic kidney failure	52 (12.5)
	Lymphoma	31 (7.4)
	Malignant tumor	43 (10.3)
Medication, *n* (%)	Antiplatelets	73 (17.5)
	Anticoagulants	63 (15.1)
	NSAIDs	109 (26.1)
	Prednisolone	53 (12.7)

Results are described with *n* (%).

The cohort of patients with liver cirrhosis included 23 patients classified as Child‐Pugh class A, 16 patients as class B, and 1 patient as class C. The mean Child‐Pugh score was 6.4.

Abbreviation: NSAIDs, non‐steroidal anti‐inflammatory drugs.

### Initial CE and treatment results

**FIGURE 1:**
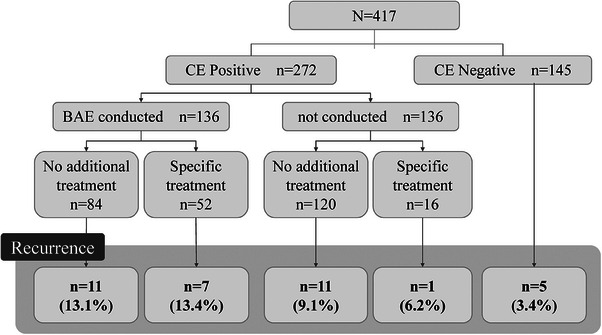
Patient flowchart. BAE, balloon‐assisted endoscopy; CE, capsule endoscopy.

Whole small intestine observation was possible in 94%.　CE revealed findings in 272 cases (65.2%). Of these, 97 patients (35.7%) had mucosal breaks, vascular lesions in 79 patients (29%), tumors or submucosal tumors (SMTs) in 22 patients (8.1%), and sources of bleeding outside the small intestine in 34 patients (12.5%). In contrast, 145 patients (34.7%) exhibited no significant findings (CE negative; Table 3). Among the patients with CE‐positive findings, BAE was conducted in 136 patients (50%). The therapeutic intervention was carried out in 52 patients, including 34 instances of endoscopic hemostasis (32 of clipping and two of endoscopic mucosal resection), 11 patients of surgical treatment (seven for tumor bleeding, two for Meckel's diverticulum, two for multiple vascular lesions that are difficult to treat endoscopically), and medication adjustments in seven patients (three for analgesic discontinuation and four for mucosal protectants). Eventually, in 52 patients (38.2%) where BAE was performed, treatment was administered. However, in 84 patients, although BAE was conducted, the lesions identified by CE could not be found, and no hemostatic treatment was applied. Conversely, in 136 patients with positive CE findings, BAE was not performed; however, in 16 patients, therapeutic intervention was performed with other than BAE: Argon plasma coagulation (APC) for vascular lesions in the terminal ileum with colonoscopy in one patient, surgical treatment for jejunal cancer in one patient, and oral medication adjustment in 14 patients. The success rate was found to be 88.2% (Figure 1).

**TABLE 3:** Capsule endoscopy findings.

**Negative, *n* (%)**	145 (34.7)
**Positive, *n* (%)**	272 (65.2)
Mucosal break	97 (35.7)
Vascular lesion	79 (29.0)
Tumor	22 (8.1)
Other findings	4 (1.5)
Only blood	36 (13.2)
Other organs^*^	34 (12.5)

Abbreviations: Negative, negative capsule endoscopy findings.; Positive, positive capsule endoscopy findings.

* Other organs include the following:　Stomach: eight cases, Duodenum: 13 cases, Colon: 13 cases.

### Long‐term course and recurrence predictors

**FIGURE 2:**
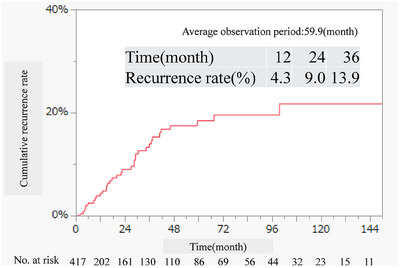
The Kaplan‐Meier analysis regarding the cumulative relapse rate.

The median follow‐up period was 8.46 months (range 1.19–50.3 months), with 35 patients (8.4%) experiencing a recurrence. The cumulative recurrence rates at 12, 24, and 36 months were 4.3%, 9.0%, and 13.9%, respectively (Figure 2). Of the recurrence cases, 30 patients had initial CE‐positive findings, and five had CE‐negative findings. The specifics of the 29 patients with positive initial CE findings related to bleeding are depicted in Figure 1. At the time of recurrence, symptoms included hematochezia or melena in 13 patients and progressive anemia in 22 patients. Recurrence risk factors were analyzed using Cox proportional hazards analysis. We included the following specific criteria: age, sex, OGIB type, current comorbidities such as liver cirrhosis and chronic kidney disease, use of the antithrombotic drug, an anticoagulant drug, and endoscopic findings of mucosal break, vascular lesion, and negative findings. In the univariate analysis, factors such as liver cirrhosis (HR: 4.34, *p* < 0.01), vascular lesions (HR: 3.14, *p* < 0.01), and negative CE findings (HR: 2.79, *p* = 0.03) were identified. Multivariate analysis revealed that only liver cirrhosis (HR: 4.15, *p* < 0.01) was a significant predictive factor. Gender, age, oral medication use and therapeutic interventions were not significant predictors of recurrence (Table 4).

**TABLE 4:** Predictive factors of relapse.

	Univariate		Multivariate	
Variables	HR (95% CI)	*p*‐value	HR (95% CI)	*p*‐value
Age				
≤60	1.35 (0.66–2.74)	0.39	1.69 (0.78–3.65)	0.17
>60	1		1	
Sex				
Female	1.11 (0.56–2.17)	0.76	1.20 (0.59–2.43)	0.61
Male	1		1	
OGIB type				
Overt	1.31 (0.66–2.59)	0.42	1.10 (0.53–2.27)	0.79
Occult	1		1	
LC				
Present	4.34 (2.21–8.55)	<0.01	4.15 (1.88–9.18)	<0.01
Absent	1		1	
CKD				
Present	1.15 (0.47–2.78)	0.75	1.05 (0.41–2.56)	0.95
Absent	1		1	
Anticoagulant use				
Yes	1.28 (0.56–2.95)	0.55	1.27 (0.52–3.10)	0.58
No	1		1	
Antiplatelet use				
Yes	1.03 (0.43–2.49)	0.94	1.83 (0.71–4.66)	0.21
No	1		1	
Mucosal break				
Present	1.07 (0.43–1.99)	0.86	1.05 (0.36–3.08)	0.92
Absent	1		1	
Vascular lesion				
Present	3.14 (1.6–6.14)	<0.01	1.82 (0.66–5.02)	0.24
Absent	1		1	
Negative findings of CE				
Yes	2.79 (1.08‐7.21)	0.03	2.05 (0.58–7.17)	0.26
No	1		1	

Abbreviations: CI, confidence interval; CKD, chronic kidney disease; HR, hazard rate; LC, liver cirrhosis; OGIB, obscure gastrointestinal bleeding.

Out of 40 patients with LC, 31 cases had positive CE (Table 5). Among them, 14 cases had a recurrence, and 13 cases had positive CE (Table 6).

**TABLE 5:** Capsule endoscopy findings in liver cirrhosis.

	Total	Child–Pugh A	Child–Pugh B	Child–Pugh C
***N* (specific treatment)**	**40**	**23** ^5^	**16 (0)**	**1 (0)**
**Negative (specific treatment), *n***	**9 (0)**	**5 (0)**	**4 (0)**	**0 (0)**
**Positive (specific treatment), *n***	**31** ^5^	**18** ^5^	**12 (0)**	**1 (0)**
Mucosal break	8^1^	5^1^	3 (0)	0
Vascular lesion	18^3^	10^3^	7 (0)	1 (0)
Only blood	2^1^	2^1^	0 (0)	0
Other organs^*^	3 (0)	1 (0)	2 (0)	0

* Other organs include the following:　Stomach: three cases.

**TABLE 6:** Recurrence rate in liver cirrhosis.

	Total	Child–Pugh A	Child–Pugh B	Child–Pugh C
***N* (specific treatment)**	**14** ^2^	**10** ^2^	**4 (0)**	**0 (0)**
**Negative (specific treatment), *n***	**1 (0)**	**1 (0)**	**0 (0)**	**0 (0)**
**Positive (specific treatment), *n***	**13** ^2^	**9** ^2^	**4 (0)**	**0 (0)**
Mucosal break	3 (0)	2 (0)	1 (0)	0 (0)
Vascular lesion	8^2^	6^2^	2 (0)	0 (0)
Only blood	1 (0)	1 (0)	0 (0)	0 (0)
Other organs^*^	1 (0)	0 (0)	1 (0)	0 (0)

* Other organs include the following: Stomach: three cases.

## DISCUSSION

In this study, we focused on the cumulative recurrence rate and risk factors associated with recurrence following the initial CE in patients with OGIB. Evaluating the recurrence rate of OGIB is critical for enhancing clinical management strategies. Our findings indicate that 16.3% of the patients exhibited positive CE results, enabling therapeutic intervention. Moreover, the overall recurrence rate of OGIB was determined to be 8.2%, with liver cirrhosis identified as a particularly significant risk factor for recurrence. Based on the results presented in Figure 2, we believe that careful follow‐up for at least 48 months is necessary to adequately monitor for recurrence.

Several studies have investigated the rebleeding rates of OGIB, yielding diverse outcomes. Niikura et al.^13^ conducted a retrospective analysis of 320 patients undergoing CE for OGIB, reporting a mean patient age of 65 years. Among these, 46% had overt OGIB, and 29% demonstrated positive CE findings, such as erosions, ulcers, tumors, and telangiectasias, with 7.5% undergoing therapeutic intervention post‐CE. The study reported a rebleeding rate of 13.4% over an average follow‐up of 18.3 months, with cumulative rebleeding rates of 11% at 12 months and 18.5% at 36 months. Conversely, Panu et al.^14^ assessed 173 patients with OGIB via CE, finding a mean patient age of 65.4 years, with 60.4% presenting with overt OGIB and 42.1% showing positive CE findings. Therapeutic intervention was performed in 26.3% of patients. The study observed a rebleeding rate of 26.3% during an average 26‐month observation period. In our study, we observed that the relapse rates for patients who did not receive specific treatment after BAE and those who received specific treatment were nearly identical. However, it is crucial to recognize that our analysis focuses on short‐term outcomes. The potential benefits of specific treatment for small bowel lesions may become more apparent over a longer period.

A comprehensive analysis of these findings, alongside our study results, suggests an approximate recurrence rate of 10–30%. The variable recurrence rates can be attributed to the broad range of OGIB causes, complicating accurate diagnosis and treatment. Study design, patient demographics, and follow‐up duration also contribute to these variations.

Furthermore, several studies have identified various risk factors for rebleeding, with cirrhosis consistently reported as a significant risk factor.^14^, ^15^, ^16^ The increased recurrence risk in liver cirrhosis patients is attributed to venous system stasis in the small intestine due to elevated portal pressure and reduced blood clotting capabilities stemming from lowered platelet counts and impaired liver function. If gastrointestinal bleeding occurs, these conditions can exacerbate bleeding or cause portal hypertensive gastroenteropathy, further compromising blood vessel integrity throughout the gastrointestinal tract.^17^ Additional reported risk factors for rebleeding include vascular lesions,^18^, ^19^ overt OGIB,^15^, ^19^, ^20^ duration of OGIB^21^ older age,^22^, ^23^, ^24^ bleeding from the jejunum,^22^ and a history of blood transfusions,^20^, ^24^ although these factors were not significant in our study. Tojo et al.^25^ have reported a potential association between Saurin classification P2 and rebleeding. The study found that P2 findings on CE were more frequent in patients taking antiplatelet drugs. Among the studies that have proposed models for predicting rebleeding in OGIB^26^, the RHEMITT score,^27^ which has been validated and has been shown to be reliable, is the most used model. This score is calculated from seven variables: renal disease, heart failure, endoscopic capsule lesion, major bleeding, incomplete capsule, tobacco, and treatment by endoscopy. The score is calculated from the following seven variables However, the RHEMITT score does not include cirrhosis, which was a risk factor for rebleeding in this study and in other predicting models for rebleeding.^13^, ^28^, ^29^ The inclusion of additional risk factors may further improve the accuracy of the predicting model for rebleeding. High‐risk patients require cautious management to mitigate the likelihood of recurrence.

While accurate diagnosis and treatment of OGIB are fundamentally crucial for reducing recurrence risk, advancements in OGIB diagnosis have outpaced treatment research.^30^ Currently, endoscopic treatment, surgical interventions, and radiotherapy are the common treatment modalities for OGIB. In surgical treatment, it shows excellent results in individual lesions such as tumors and localized arteriovascular malformations. However, lesions that are used more extensively, such as multifocal angioectasia as in our case, are usually treated endoscopically at the time of surgery. Although rebleeding rates are expected to be similar between surgical treatment and endoscopic hemostasis, long‐term follow‐up data are not available.^6^ In radiotherapy, the advantage of angiography is that if the source of bleeding is identified during diagnosis using angiography, it can be treated with embolization, and unlike endoscopy, there is no difficulty in obtaining a visual field due to bleeding. Therefore, angiography is considered to be indicated when endoscopic hemostasis is difficult due to active bleeding. However, in our study, there were no cases in which endoscopic hemostasis was difficult and the patient underwent angiography.^6^ Endoscopic options include clipping for hemostasis, APC, and endoscopic band ligation, with treatment selection based on lesion location, bleeding severity, and patient comorbidities. Despite the absence of large‐scale prospective studies comparing treatment efficacy, various treatments have been explored, including hormonal therapies, somatostatin analogs, thalidomide, erythropoietin, and Von Willebrand factor. However, these pharmacological treatments have yet to demonstrate definitive efficacy and are primarily considered for patients who continue to bleed post‐endoscopic treatment or those ineligible for surgical intervention.^31^ However, supportive therapy with oral or intravenous iron is said to be the only primary means of treatment for minor small bowel bleeding.^32^ Given the challenges in managing portal hypertension and its sequelae in liver cirrhosis, regular monitoring and early detection of recurrence signs are important in patients with comorbidity of liver cirrhosis, including other risk factors elevating the risk of OGIB recurrence as mentioned above. Future efforts should focus on refining treatment and management strategies for OGIB.

This study has some limitations. A retrospective nature and reliance on data from a single academic center potentially have introduced selection bias. In addition, the criteria for additional examinations or treatments after CE could vary based on historical background. Dependence on medical records and external referrals for patient follow‐up may overlook certain recurrence instances, possibly underestimating the recurrence rate. Further research is needed to evaluate the long‐term efficacy and impact of specific treatments on recurrence rates. Saurin classification should normally be used for vascular lesions, but this was not done in this case because there were old cases and there was insufficient information to classify them.

In conclusion, this study reports an OGIB recurrence rate of 8.2%, with a 3‐year cumulative recurrence rate of approximately 14%, and underscores liver cirrhosis as a risk factor for recurrence. Despite the challenges in diagnosing and treating OGIB, diligent follow‐up is essential, particularly for patients with liver cirrhosis. Future research should be conducted with large‐scale prospective studies to further understand and address OGIB management.

## CONFLICT OF INTEREST STATEMENT

None.

## ETHICS STATEMENT

This study was conducted according to the ethical guidelines of the Declaration of Helsinki and was approved by the Institutional Review Board of Gifu University Hospital. Registry and the Registration　N/A. Animal Studies. N/A.

## PATIENT CONSENT STATEMENT

The opt‐out method was used to obtain consent from study participants.
